# Characterization of a Novel Mitochondrial Ascorbate Transporter From Rat Liver and Potato Mitochondria

**DOI:** 10.3389/fmolb.2018.00058

**Published:** 2018-06-26

**Authors:** Vito Scalera, Nicola Giangregorio, Silvana De Leonardis, Lara Console, Emanuele Salvatore Carulli, Annamaria Tonazzi

**Affiliations:** ^1^Department of Bioscience, Biotechnology and Biopharmaceutics, University of Bari, Bari, Italy; ^2^CNR-IBIOM (Institute of Biomembranes, Bioenergetics and Molecular Biotechnologies), Bari, Italy; ^3^Department of Biology, University of Bari “Aldo Moro,”, Bari, Italy; ^4^Unit of Biochemistry and Molecular Biotechnology, Department DiBEST (Biologia, Ecologia, Scienze della Terra), University of Calabria, Rende, Italy

**Keywords:** ascorbic acid, mitochondria, transport, liposomes, reconstitution, plants

## Abstract

The Mitochondrial Ascorbic Acid Transporter (MAT) from both rat liver and potato mitochondria has been reconstituted in proteoliposomes. The protein has a molecular mass in the range of 28–35 kDa and catalyzes saturable, temperature and pH dependent, unidirectional ascorbic acid transport. The transport activity is sodium independent and it is optimal at acidic pH values. It is stimulated by proton gradient, thus supporting that ascorbate is symported with H^+^. It is efficiently inhibited by the lysine reagent pyridoxal phosphate and it is not affected by inhibitors of other recognized plasma and mitochondrial membranes ascorbate transporters GLUT1(glucose transporter-1) or SVCT2 (sodium-dependent vitamin C transporter-2). Rat protein catalyzes a cooperative ascorbate transport, being involved two binding sites; the measured K_0.5_ is 1.5 mM. Taking into account the experimental results we propose that the reconstituted ascorbate transporter is not a GLUT or SVCT, since it shows different biochemical features. Data of potato transporter overlap the mammalian ones, except for the kinetic parameters non-experimentally measurable, thus supporting the MAT in plants fulfills the same transport role.

## Introduction

Ascorbate is a multifaceted ubiquitous, small molecular weight, carbohydrate derivative that plays important roles in several cellular processes in plant and animal cells, being cofactor of many enzymes and involved in antioxidant cellular protection (Englard and Seifter, 1986; Patak et al., 2004; Mandl et al., 2009; Padayatty and Levine, 2016). Ascorbic acid is synthesized in vegetal cells and in some mammals, but it is a vitamin for others such as humans (Lachapelle and Drouin, 2011). In plants, beyond these physiological functions, ascorbate is required in responses to abiotic stresses, such as high salinity, high light or drought (Bandurska et al., 2013). Mammals cells take up ascorbic acid by two high affinity/low capacity Na^+^-dependent transporters: SVCT1 (SLC23A1) and SVCT2 (SLC23A2), both constituted of about 600 aminoacids and with similar hydropathic profiles predicting 12 putative membrane-spanning domains, with different tissue distribution (Tsukaguchi et al., 1999). SVCT1 is expressed in epithelial tissues, contributing to the maintenance of whole-body vitamin C levels, whereas the expression of SVCT2 is relatively widespread (Cantoni et al., 2017). It has also been demonstrated that the oxidized form of ascorbate, dehydroascorbic acid (DHA), is taken up into mammalian cells by members of hexose transporters family GLUTs, and in the cytosol it is enzymatically reduced to ascorbic acid (Rumsey et al., 1997; Corti et al., 2010; Bánhegyi et al., 2014). Plant cells are able to synthesize ascorbate, mostly in photosynthetic tissues, which is transported through the phloem from source to sink organs, such as flowers and developing tubers (Tedone et al., 2004). Some studies have proposed the presence of specific plasma membrane transporters for ascorbic acid or DHA in plants (Horemans et al., 2000) but the molecular identity of the transport proteins have not still been well elucidated (Venkatesh and Park, 2014). Cytosolic vitamin C in mammals and plants is in part directed to sub-cellular compartments such as peroxisomes, mitochondria, chloroplasts, where, beyond its role as cofactor, is fundamental for its involvement in red-ox homeostasis and is part of oxidative signaling since, together with other antioxidants as glutathione, hampers ROS signals (Foyer and Noctor, 2005; Tonazzi et al., 2013, 2017; Bánhegyi et al., 2014; Venkatesh and Park, 2014). In spite of wider knowledge on the plasma membrane transporters there are still controversial information concerning the transport mechanism of vitamin C in mitochondria (Cantoni et al., 2017). It has been demonstrated that ascorbate concentration in mammalian mitochondria can be increased by dietary vitamin C supplementation and its concentration varies according to metabolic conditions (Li et al., 2001). It has been proposed that DHA is produced in selective micro domains close to the respiratory chain, on the external side of the inner mitochondrial membrane, both in mammals and plants, and is then transported across the mitochondrial membrane by the high capacity hexose transporter GLUT1 (Szarka et al., 2004; KC et al., 2005). The paramount advances in clarifying its role in mitochondria were the discovery of the link between the mitochondrial electron transport chain and ascorbate biosynthesis (Bartoli et al., 2000). In the mitochondrial matrix DHA is reduced to ascorbate by electrons generated by the complex III of the respiratory chain and by DHA reductase (Li et al., 2002; Gallie, 2013). In plant mitochondrial matrix DHA is reduced to ascorbic acid also by ascorbate free radical reductase (AFR) (De Leonardis et al., 1995) while in mammalian mitochondria, by a GSH dependent mechanism or by lipoic acid and mitochondrial TrxR (Li et al., 2001). Recently it has been demonstrated that human HEK-293 cells express a mitochondrial ascorbic acid transporter (MAT) that kinetically corresponds to a low-affinity form of the sodium-coupled ascorbic acid transporter-2 (SVCT2) (Muñoz-Montesino et al., 2014), also responsible of ascorbate transport in U937 cell mitochondria and whose activity is proposed to be regulated by DHA (Fiorani et al., 2014, 2015; Cantoni et al., 2017). Most of the data concerning mammalian ascorbate transport have been obtained in intact cells and intact mitochondria studies. These studies give fundamental information on mitochondrial ascorbate transport but are indeed affected by some limitations and interferences related with intact cells studies.Cultured cells are, for instance associated with an altered oxidative stress due to the about 10 times higher oxygen tension than *in vivo* which may interfere with ascorbate cellular equilibrium (Cantoni et al., 2017). This paper is focused on the “*in vitro*” biochemical characterization of the MAT. Following a procedure that has been used for several mitochondrial transporters (Palmieri et al., 1995; Palmieri, 2013), a protein which catalyzes the ascorbic acid transport, has been isolated from rat liver and potato mitochondria, reconstituted in proteoliposomes and functionally characterized.

## Materials

Sephadex G-75 was purchased from Pharmacia, Hydroxyapatite (HTP) from Bio-Rad, L-[1-^14^C]Ascorbic acid (Product Number: NEC146), specific activity range 2–10 mCi/mmol (74–370 MBq/mmol) from PerkinElmer, Inc., egg-yolk phospholipids (l-α-phosphatidylcholine from fresh turkey egg yolk) and Amberlite XAD-4 from Fluka, ascorbic acid, dehydroascorbate (DHA), HEPES, Triton X-100, cardiolipin, HgCl_2_, pyridoxal phosphate from Sigma, St. Louis, MO. All other reagents were of analytical grade.

### Methods

#### HTP chromatography of mitochondrial extracts

The animal experiments were performed according to the European Community guidelines for care and use of animals. In particular animals were housed in air conditioned room with 12/12 h dark–light cycle, with food and water *ad libitum*, in the approved facility at the University of Bari. All animal procedures were performed in accordance with the Guidelines for Care and Use of Laboratory Animals of the “University of Bari (OPBA di Ateneo)” and the Italian Ministry of Health. Adult male Sprague-Dawley rat (age of 6–12 months) liver mitochondria were prepared as described in (Johnson and Lardy, 1967). Highly purified mitochondrial fractions devoid of mitochondrial associated membranes (MAMs) were obtained after fractionation by Percoll 30 % gradient (Wieckowski et al., 2009). Potato mitochondria, with a high yield of purification from other cellular components, were prepared according to Salvato et al. (2014). The average purity of the mitochondrial preparation was monitored by measuring the specific activity of alkaline phosphatase (EC 3.1.3.1). The specific activity of alkaline phosphatase, in the mitochondria preparations was measured, at 37°C, as substrate consumed per min per mg protein (Scalera et al., 1980). Mitochondria either from rat liver (40–60 mg protein/ml) or from potato (10–20 mg protein/ml) were solubilized in 3% Triton X-100, 20 mM Na_2_SO_4_, and 10 mM Pipes pH 7 (solubilization buffer), and centrifuged at 100,000 × g for 15 min at 4°C. Mitochondrial extract, 0.5 ml, were applied onto a dry hydroxyapatite column (0.4 g HTP, 0.5 cm diameter) and eluted with the solubilization buffer. The first two fractions of 0.5 ml each of eluate were collected (hydroxyapatite eluate), these procedures were performed at 4°C.

#### Reconstitution of the ascorbate transporter in liposomes

The mitochondrial extracts or the hydroxyapatite eluates were reconstituted by the detergent removal method, using a column of hydrophobic resin Amberlite XAD-4 (Tonazzi et al., 2015; Scalise et al., 2017). In this procedure, the mixed micelles containing detergent, protein and phospholipids were repeatedly passed through the same Amberlite column (3.5 cm × 0.5 cm). The resin is highly affine for hydrophobic molecule and, at each passage of the reconstitution mixture, gradually removes the detergent molecules allowing the proteoliposomes assembly. The composition of the starting mixture used for reconstitution was: 75 μl extract and 90 μl 10% Triton X-100 or 250 μl HTP eluate and 50 μl 10% Triton X-100; 120 μl, 10% egg yolk phospholipids, in the form of sonicated liposomes; 5 mM L-ascorbate or 5 mM DHA when present; 20 mM HEPES/TRIS pH 6.5 where not differently specified; water was added to reach a final volume of 680 μl. After vortexing, the mixture was passed 14 times through the same Amberlite column, equilibrated with 20 mM of the same buffer used in the reconstitution mixture. All the operations were performed at 4°C, except the passages through Amberlite which were performed at room temperature.

#### Transport measurements

In order to remove the external salts and substrate, when present, 550 μ1 proteoliposomes were passed through a Sephadex G-75 column (0.7 cm × 15 cm), equilibrated with 10 mM HEPES/TRIS, pH 6.5 where not differently indicated. The eluted proteoliposomes (600 μl), distributed in reaction vessels (100 μl), were used for transport measurements using the inhibitor-stop method (Palmieri et al., 1995). Transport was started by adding 10 μl 1.1 mM [^14^C]ascobate (final concentration 0.1 mM) and stopped after the indicated times by adding 5 μ1 300 mM PLP. In control samples, the inhibitor was added together with the radiolabeled substrate. The assay temperature was 25°C. In order to remove the radioactivity not taken up by the proteoliposomes, 100 μl of each sample were passed through a Sephadex G-75 column (0.6 cm × 8 cm). The proteoliposomes were eluted with 1.2 ml 50 mM NaC1 and collected in 3 ml scintillation mixture, vortexed, and counted. The transport activity was evaluated as the difference between the experimental and the control values from measurements performed after the incubation time. For kinetic experiments, initial transport rate was derived from the isotope equilibration kinetics and stopping the transport reaction after different time intervals (10 and 40 min). Data fitted according to a first-order process equation thus allowing the initial transport rate to be calculated, expressed in nmol × mg protein^−1^ × min^−1^.

#### Other method

Protein inactivation was obtained incubating 50 μg/ml of trypsin for 3 h at 25°C or boiling the protein for 20 min. Polyacrylamide slab-gel electrophoresis was performed in the presence of 0.1% SDS. A minigel system was used; gel sizes were 8 cm × 10 cm × 0.75 mm (thickness). The stacking gel contained 5% acrylamide and the separation gel contained 17.5% acrylamide and an acrylamide/bisacrylamide ratio of 30:0.2. Staining was performed by the Blue Comassie method. Protein was determined using the Bio-Rad Protein assay.

## Results

### Enrichment of ascorbate transporter from mitochondrial extracts by HTP chromatography

**Figure 1:**
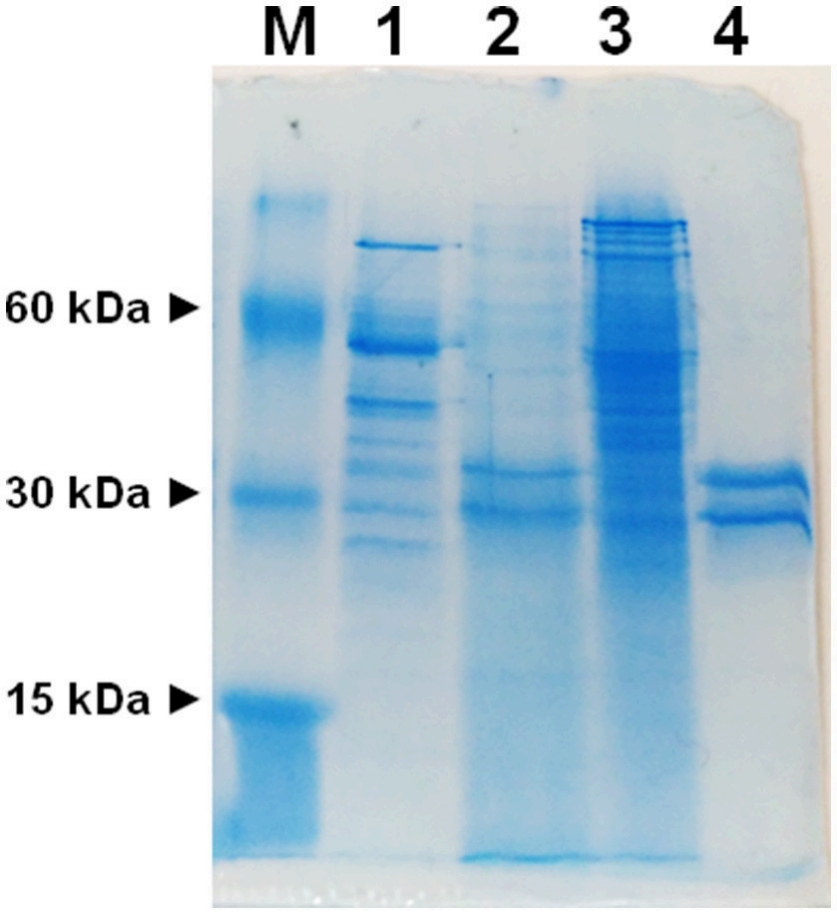
SDS-PAGE of mitochondrial extracts and HTP chromatography eluates. M, marker proteins (bovine serum albumin, carbonic anhydrase and cytochrome c); (1) potato mitochondrial extract (12.6 μg), (2) potato mitochondrial hydroxyapatite eluate (65 μg), (3) liver mitochondrial extract (42 μg), (4) liver mitochondrial hydroxyapatite eluate (25 μg).

To avoid interferences, related with the presence of plasma membrane transporters, highly purified mitochondria were obtained according to optimized procedures that have been used also for proteomic analyses (Wieckowski et al., 2009; Salvato et al., 2014). To asses mitochondrial purity Alkaline Phosphatase (AP) activity was measured. AP is a marker enzyme for the plasma membrane and it was determined both in the tissue homogenate and in the final mitochondrial preparation. The AP relative activities calculated (i.e., specific activity in purified mitochondria/specific activity in homogenate) were: 0.2 in rats and 0.3 in potato showing a low plasma membrane contamination. Mitochondria, isolated from rat liver and from potato, were solubilized using Triton X-100, as described in Methods. The protein extracts were reconstituted in the liposomes using the detergent removal method in the absence of internal substrate. Ascorbic acid transport activity was detected in both rat liver and potato protein extracts, being 0.027 nmol × mg protein^−1^ × 10 min^−1^ and 0.12 nmol × mg protein^−1^ × 10 min^−1^ respectively (Table 1). The mitochondrial extract, 500 μl, was passed through a column loaded with dry hydroxyapatite resin, see Methods. The first 500 μl of HTP eluate were collectedand reconstituted, see Methods. Specific activity of rat liver HTP eluate was 1.1 nmol × mg prot^−1^ × 10 min^−1^ and of potato HTP eluate 0.25 nmol × mg prot^−1^ × 10 min^−1^. It was also collected and reconstituted a second HTP fraction of 500 μl but no transport activity was detected. As evidenced in Table 1 and Figure 1 a partial enrichment of the ascorbic acid transporter was achieved after HTP chromatography, being higher for the rat protein. The proteins from rat liver and potato mitochondria were respectively 40 and 2 fold more concentrated with respect to the mitochondrial extracts (Table 1). The pattern of the SDS/PAGE shows that after HTP chromatography most of the proteins, present in the mitochondrial extracts (Figure 1, lanes 1 and 3), bind to the resin since only few protein bands are evident in the hydroxyapatite eluate of both rat liver and potato (Figure 1, lanes 2 and 4, HTP first eluate). The protein bands are in the range of 30 KDa. Since cardiolipin has been successfully used to improve the purification and/or the transport activity of several mitochondrial transport proteins, the effect of this lipid on the ascorbic acid transport was analyzed. The unidirectional ascorbic acid transport was indeed insensitive to the presence of cardiolipin during the solubilization or during the reconstitution procedure (not shown). The addition during the solubilization and HTP chromatography and/or the reconstitution procedure of the strong reducing reagent DTE, that in some cases activates transport activity of reconstituted mitochondrial carriers (Tonazzi et al., 2005), also didn't affect the transport of ascorbate in the reconstituted system.

**Table 1:** Partial protein enrichment of the mitochondrial ascorbic acid transporter from rat liver and potato.

		**Protein (mg/ml)**	**Specific activity**	**Protein enrichment (fold)**
Rat	Extract	28.5	0.027 ± 0.0042	–
	HTP	0.49	1.1 ± 0.22	40
Potato	Extract	8.4	0.12 ± 0.031	–
	HTP	1.3	0.25 ± 0.017	2.0

*The uptake was started by adding 0.1 mM external [^14^C]ascorbic acid to the proteoliposomes in the absence of internal substrate. Other conditions as described in Methods. The activity of the reconstituted ascorbic acid transporter is expressed in nmol × mg protein^−1^ × 10 min^−1^ (specific activity)*.

### Characterization of ascorbic acid transport in the proteoliposome reconstituted system

**Figure 2:**
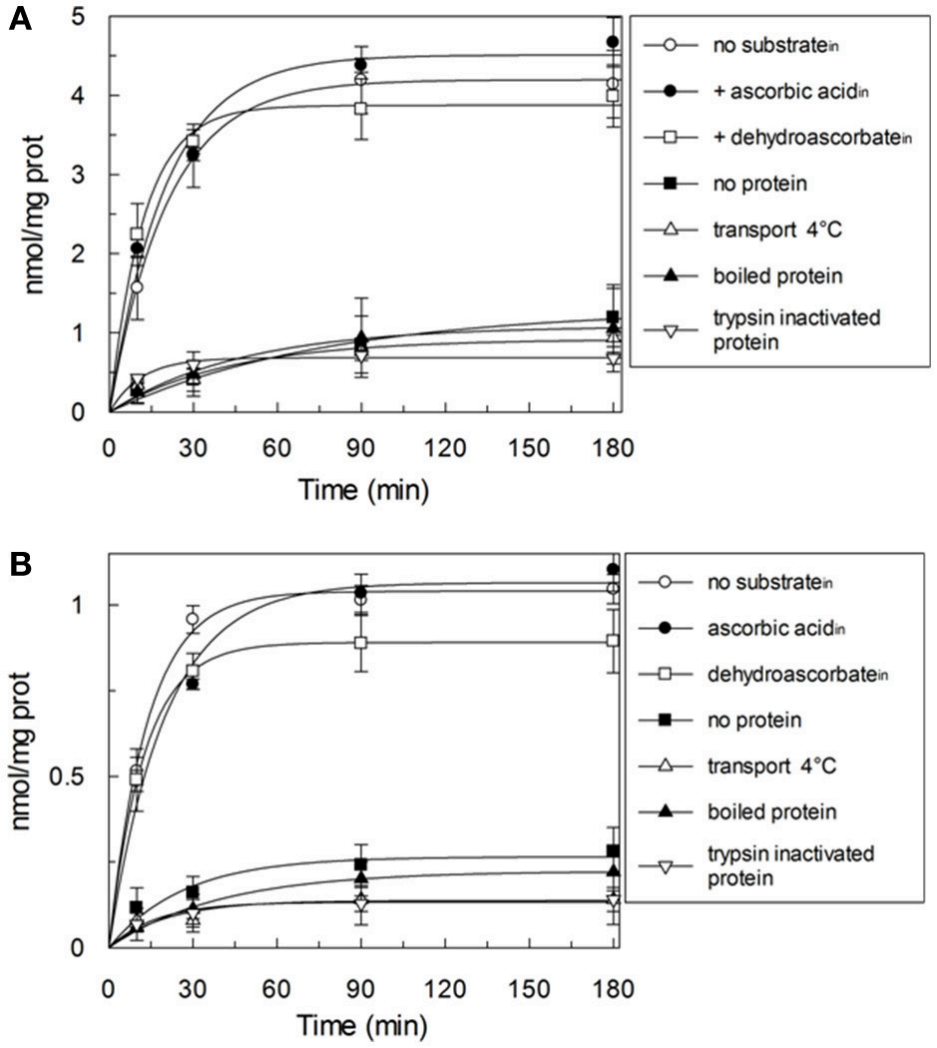
Time-course of the ascorbic acid uptake in reconstituted proteoliposomes. The uptake was started by adding 0.1 mM external [^14^C]ascorbic acid at time zero to proteoliposomes reconstituted with HTP eluate from rat liver **(A)** or potato **(B)**. Proteoliposomes were reconstituted without (◦,■,Δ,▴,∇) or with 10 mM ascorbic acid (•) or 10 mM DHA (□) as internal substrate. Transport was stopped by adding 15 mM PLP after the indicated time intervals. Data are means ± SD from three independent experiments.

HTP eluates from rat liver and potato mitochondria were reconstituted and the effect of various inhibitors on the ascorbic acid uptake, was investigated. Transport was efficiently, even though not completely, inhibited only by PLP (80%), while phenylmaleimmide, p-hydroxymercuribenzoate and bathophenanthroline partially reduced the transport of ascorbic acid. Both rat and potato transporters were insensitive to HgC1_2_, N-ethylmaleimide, 5,5′-dithiobis(2-nitrobenzoate) (DIDS). Also the known inhibitors of GLUT transporters, D galattosammine, and cytochalasin B did not exert any effect against the transport of ascorbic acid, as well as mannitol and glucose (Table 2). Figure 2 shows a time dependence of labeled ascorbic acid uptake into proteoliposomes reconstituted with HTP eluate from rat liver, Figure 2A, and potato, Figure 2B, mitochondria. Almost no labeled ascorbic acid uptake was detected into proteoliposomes reconstituted with boiled or trypsin inactivated protein or without protein. The activity was negligible also if the transport was measured at 4°C. These results sustain that the observed transport is protein mediated and not ascribable to aspecific radiolabelled substrate binding or uptake into proteoliposomes. As shown in (Figure 2) the uptake of [^14^C]ascorbic acid into the proteoliposomes containing intraliposomal ascorbic acid or DHA or reconstituted without internal substrate, was nearly overlapping, highlighting that it is an unidirectional uptake. The radioisotopic equilibrium was reached after about 60 min of incubation. Similar results were obtained both from rat and potato proteins (Figure 2). These data indicate that the transport process occurred by a uniport mode. The experimental data fitted a first order rate equation and the rate constant (*k*) calculated for the unidirectional transport was: 0.048 min^−1^ ± 0.0016 min^−1^ for rat and 0.073 ± 0.0035 min^−1^ for potato. The transport rate (calculated as the product of *k* × transport at equilibrium) was 0.21 ± 0.042 nmol × mg protein^−1^ × min^−1^ for rat and 0.076 ± 0.0041 nmol × mg protein^−1^ × min^−1^ for potato. The effect of sodium and calcium was tested on the ascorbate transport. These experiments were performed using the reconstituted mitochondrial extracts instead of HTP eluate to avoid interferences from traces of salts deriving from the resin. Transport was performed in the absence or in the presence of CaCl_2_ 0.1 mM or of NaCl 20 mM. It was evident that the transport of ascorbic acid was independent from the presence of both calcium or sodium (not shown), thus excluding the occurrence of sodium dependent co-transport or of activation of the transport by calcium.

**Table 2:** Effect of inhibitors on the reconstituted ascorbic acid unidirectional uptake.

**Inhibitor**	**(mM)**	**Inhibition (%)**	
		**Rat**	**Potato**
N-Ethylmaleimide	2.0	0	0
HgCl_2_	0.5	0	0
4,4′-Diisothiocyano-2,2′-stilbenedisulfonic acid (DIDS)	5.0	0	0
Glucose	20	0	0
Mannitol	20	0	0
Cytochalasin B	0.5	0	0
Galactosamine	20	0	0
p-Hydroxymercuribenzoate	1.0	40 ± 7.5	38 ± 8.0
Phenylmaleimide	1.0	43 ± 8.5	46 ± 8.3
Bathophenanthroline	20	45 ± 3.5	44 ± 11
Pyridoxal 5′-phosphate (PLP)	15	80 ± 4.0	75 ± 5.3

*The uptake was started by adding 0.1 mM external [^14^C]ascorbic acid to proteoliposomes reconstituted with HTP eluate, from rat liver and from potato, in the absence of internal substrate. The inhibitors were added together with the labeled substrate. The control value of uninhibited ascorbic acid uptake was 1.13 nmol × mg protein^−1^ × 10 min^−1^ and 0.28 nmol × mg protein^−1^ × 10 min^−1^ protein respectively for rat and potato*.

### Dependence of the reconstituted ascorbic acid transport system from pH

**Figure 3:**
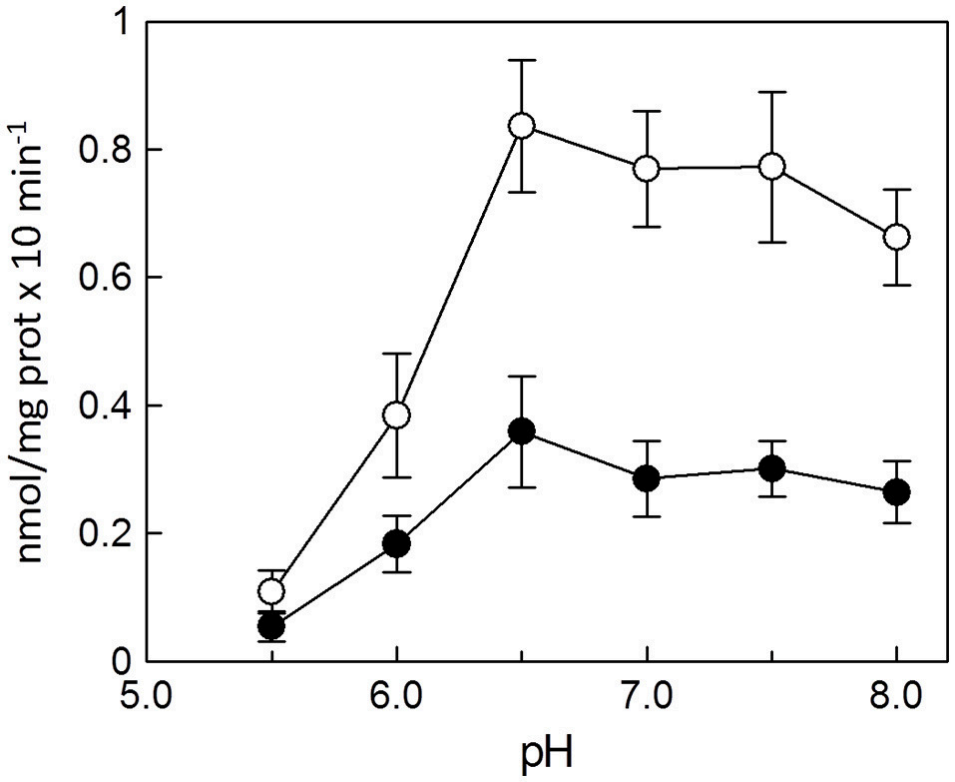
Effect of pH on the reconstituted ascorbic acid transporter from rat liver and potato. All the experimental procedures from the reconstitution to the transport measurement (see Methods) were performed in 20 mM HEPES/TRIS buffer at the indicated pH. Transport rate was measured as 0.1 mM [^14^C]ascorbic acid uptake in 30 min into proteoliposomes reconstituted with HTP eluate from rat liver (◦) or from potato (•) mitochondria. Data are means ± SD from three independent experiments.

**Figure 4:**
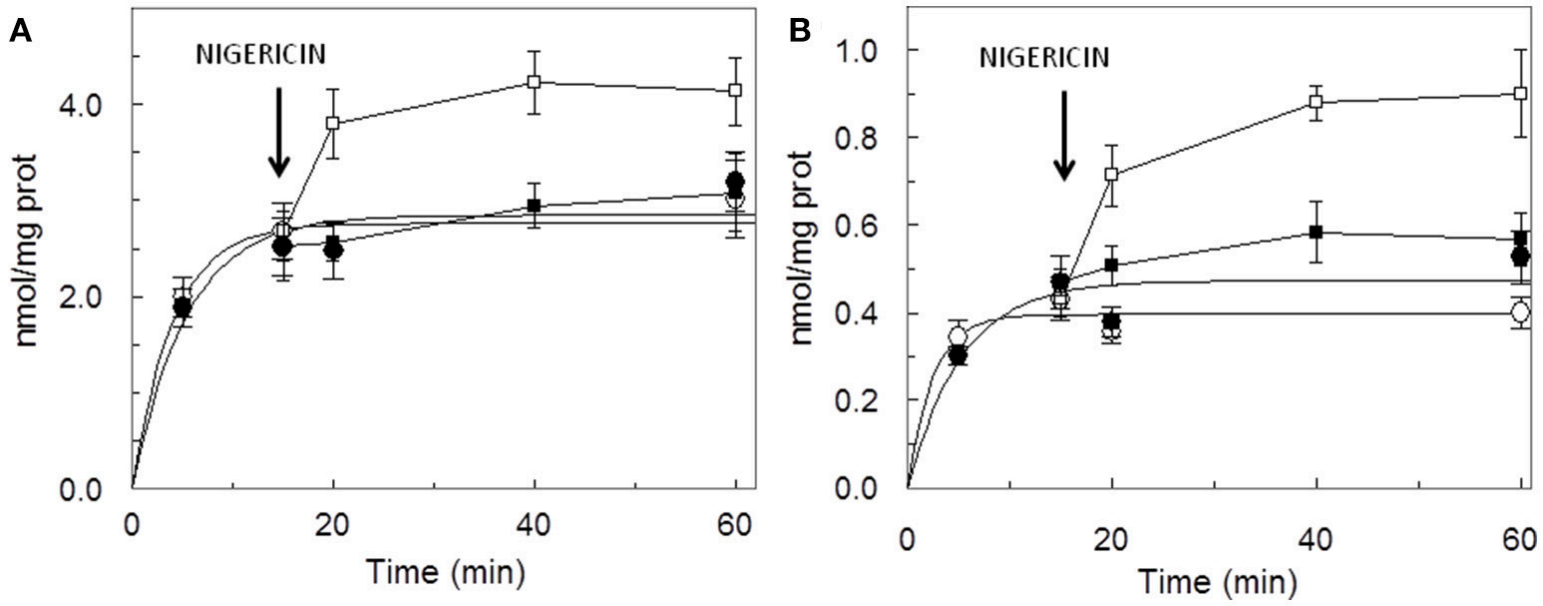
Stimulation of [^14^C]ascorbic acid entry into proteoliposomes by the pH gradient generated by the addition of nigerin. Proteoliposomes contained 50 mM NaCl, to balance osmolarity due to subsequent addition of KCl, and 0.5 mM HEPES/TRIS pH 6.5. The Sephadex G-75 equilibration and elution buffer consisted of 40 mM sucrose and 0.5 mM HEPES, pH 6.5. Transport was started by the addition of 0.1 mM [^14^C]ascorbic + 50 mM KCl (◦) or + 50 mM NaCl (•) to proteoliposomes reconstituted with HTP eluate from rat liver **(A)** or potato **(B)** and terminated at the indicated times. Nigericin (100 ng/mg of phospholipids) in ethanol (10 μl/ml of proteoliposomes) (■, □) or ethanol alone to the control samples, was added at 15 min as indicated by the arrow. Data are means ± SD from three independent experiments.

The dependence of the transport activity from the pH is reported in Figure 3. The optimal transport activity was measured at pH 6.5–7.0, for both the rat liver and potato mitochondrial proteins. The values of transport activity reported in the graph have been subtracted for the labeled ascorbic acid taken up by liposomes reconstituted without the protein and they were 0.28 ± 0.035 nmol × mg protein^−1^ × min^−1^ for rat liver and 0.12 ± 0.030 nmol × mg protein^−1^ × min^−1^ for potato. To also evaluate the influence of pH gradient on the ascorbate transport, a set of experiments were performed by generating an inwardly directed pH gradient on the proteoliposomal membrane. The delta H^+^ was created adding to the proteoliposomes the K^+^/H^+^ exchanger nigericin in the presence of 50 mM KCl_out_/no KCl_in_, i.e., imposing a K^+^ gradient. Figure 4 shows the time dependence of transport activity of rat (Figure 4A, open circles) and potato (Figure 4B, open circles) proteins in the absence of ionophore, and highlights that, after the addition of nigericin, transport activity catalyzed from both rat and potato proteins (Figures 4A,B, open squares) increases. Addition of nigericin in the absence of K^+^ gradient had no effect on the transport (Figures 4A,B, closed squares). No changes of the transport activity were evidenced when the outwardly directed proton gradient was imposed on the proteoliposomal membrane (no KCl_out_/50 mM KCl_in_), not shown.

### Kinetic parameters of the mitochondrial ascorbate transporter

**Figure 5:**
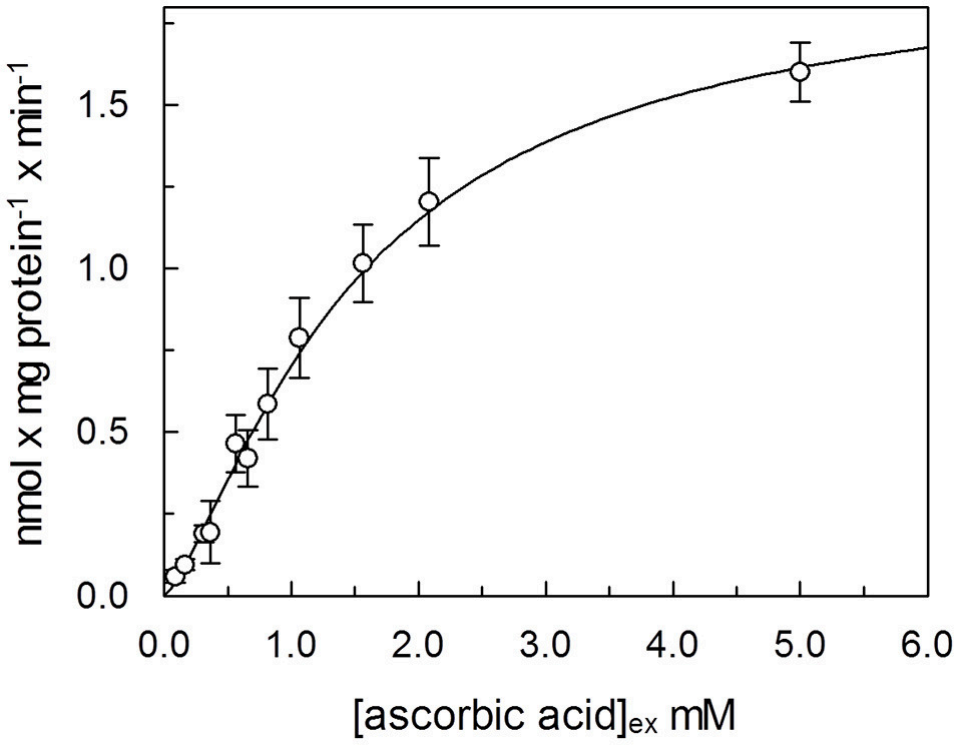
Dependence from substrate concentration of the rate of ascorbic acid transport. [^14^C]ascorbic acid, at the indicated concentration was added to proteoliposomes. The transport rate for each experimental point was calculated, as described in Methods, as product of velocity constant (*k*) × limit, derived from a time course, for each external concentration of ascorbic acid. Data were plotted according to Hill plot. Data are means of five experiments.

In order to determine kinetic constants of the unidirectional transport of ascorbic acid catalyzed by the rat liver mitochondrial transport protein, the rate of ascorbic acid uptake dependence from external substrate concentration was studied by changing the concentration of externally added [^14^C]-Ascorbic acid in the absence of intraliposomal substrate. Initial velocity values, for each ascorbic acid external concentration, were derived, as described in Methods, from a time course as the product of the first-order rate constant (*k*), and the transport at equilibrium (*limit*). The media of five experiments fitted in a Hill equation, as shown in Figure 5. The data fits a sigmoid curve, representative of cooperative binding of the substrate to the protein. The extrapolated Hill coefficient is 1.35 ± 0.071, according with the presence of two binding sites for the substrate. K_0.5_ derived from the curve is 1.5 ± 0.19 mM and Vmax 1.9 ± 0.12 nmol × mg protein^−1^. Due to methodological reasons, mainly derived from the low transport activity, kinetic parameters of the potato transport protein were not measured.

## Conclusion

In this study mitochondrial ascorbate transport has been in parallel investigated in potato and rat liver mitochondria. This comparative analysis has already been performed in several studies to unravel similarities or differences between plant and mammals mitochondrial proteins (Kesseler et al., 1992; Affourtit et al., 2001).

Both vegetal and animal mitochondria are the main ROS producing organelles thus require reducing species, such as glutathione and ascorbic acid, to hamper them. Ascorbate, in plants, is synthesized by several metabolic pathways in the cytosol and, L-Galactono-1,4-lactone dehydrogenase, the last enzyme in one of these ascorbate biosynthetic pathways is physically associated with complex I, on the external face of the inner mitochondrial membrane (Szarka et al., 2013). Ascorbate enters into mitochondrial matrix where five different enzymes use it for ROS detoxification. It has been proposed that ascorbate must be imported into mitochondria across the inner mitochondrial membrane (Salvato et al., 2014). In mammalian mitochondria ascorbate transport has been detected and attributed to SVCT meanwhile DHA transport is related to GLUT. In plant mitochondria DHA is transported by GLUT-like transporter and subsequently reduced to ascorbate (Szarka et al., 2013).

We have here, for the first time, reconstituted in proteoliposomes an enriched HTP fraction from highly purified rat liver and potato mitochondria, containing a functional ascorbic acid transporter. HTP column chromatography has been widely used to purify several mitochondria1 carriers since, due to its hydrophilic nature, binds with greater affinity soluble and glycosylated proteins and to a lesser extent hydrophobic proteins such as mitochondrial carriers (Indiveri et al., 1990, 1992; Palmieri et al., 1995). Rat and potato HTP eluates, analyzed on SDS-PAGE, highlighted a similar protein pattern, where two main protein bands in the range of 28–35 kDa are present, reflecting the overall same protein affinities for the HTP resin that led in both cases to a protein enrichment, higher for the rat liver than for potato protein. This discrepancy between mammal and plant protein reflects also on the transport activity measured in the potato HTP eluate which is indeed roughly 20% respect to rat liver HTP eluate. It can be argued that the potato transporter binds to the resin or that the chromatographic step inactivates it. The absence of higher molecular weight bands, as evident in the gel's lanes corresponding to HTP eluates, let us to exclude the presence of proteins corresponding to SVCT2 or GLUTs whose molecular masses ranges between 55 and 60 kDa. These results indicate that the protein, with ascorbate transport function, has the molecular mass and the hydrophobicity typical of the tripartite structure of mitochondrial carrier proteins (Palmieri, 2013). Proteoliposome system allowed us to study the functional properties of the transporters. A specific feature of both mammalian and plant protein is the unidirectional transport mode, that appears to be different from the common antiport mode of most mitochondrial carriers. Ascorbic acid uptake was optimal at pH 6.5–7.0 and stimulated when an inwardly directed proton gradient was generated across the proteoliposomal membrane, by the K^+^/H^+^ exchanger nigericin,. This could be ascribed to a co-transport of proton(s) or to a counter-transport of OH^−^ with ascorbate. This last mode was indeed demonstrated also for the mitochondrial phosphate carrier (Stappen and Krämer, 1994), thus corroborating that the vitamin C transporter may belong to mitochondrial carrier family. According to this interpretation we have also determined that the opposite H^+^ gradient didn't exert any effect on the transport (not shown). Of course, it cannot be completely excluded, at this stage, that the observed stimulation by H^+^ gradient may be due to H^+^ binding to functional groups of the protein outwardly exposed, i.e., a direct protein activation. The respiratory chain generates an inwardly directed proton gradient across the inner mitochondrial membrane that can support the ascorbate transport. Moreover, this also suggests that the protein is inserted in the proteoliposome membrane in the same orientation it has in the native membrane. This is a common feature of reconstituted carriers, since the ones deeply characterized are right side out inserted in the lipid belayer (Indiveri et al., 1991, 1994, 2001; Bisaccia et al., 1993). The transport activity dependence from the pH is different from GLUT1 and SVCT2 since the optimal activity for these proteins was at pH 7.5–8.0. (Tsukaguchi et al., 1999; Fiorani et al., 2015). The inhibitor sensitivity of both the rat and potato proteins has been studied and, interestingly, overlaps (Table 2). Among the tested reagents and inhibitors, the ascorbate transporter is indeed highly sensitive only to the lysine blocking reagent PLP and partially inhibited by some mercurial reagents, such as p-OHMB, and mersalyl, while was not affected by NEM, HgCl_2_ and also by DIDS, inhibitors that strongly affect SVCT2 (Luo et al., 2008). The canonical GLUT inhibitors D-galattosammine, cytochalasin B, D-glucose or D-mannitol did not inhibit the uptake of ascorbate into proteoliposomes (Rumsey et al., 1997; Szarka et al., 2004). Taken together these results allow us to exclude that the observed ascorbate transport in proteoliposomes is mediated by GLUT1 or SVCT2. Our data can also explain some discrepant results obtained from May et al. (2007) that observed ascorbate accumulation in *guinea pig* skeletal mitochondria in the presence of GLUT1 inhibitors and in absence of sodium. It was previously demonstrated that Ca^2+^ and high Na^+^ concentration are required for the optimal uptake of ascorbic acid by the plasma membrane SVCT2 (Fiorani et al., 2015), as well as that mitochondrial SVCT2 requires low (millimolar) sodium concentration and no Ca^2+^ (Muñoz-Montesino et al., 2014). Our *in vitro* data indicate that the ascorbic acid uptake into proteoliposomes, reconstituted with both rat liver and potato mitochondrial extract, were not affected by the presence of sodium, in particular we measured ascorbate transport in complete absence of sodium, highlighting that the reconstituted transporter has different features compared to plasma membrane and mitochondrial SVCT2. The kinetic analysis of ascorbate transport of the rat protein, showed a cooperative behavior, Figure 5, with an Hill coefficient of 1.35, highlighting a positive cooperative behavior and the presence of two binding sites for the substrate. The K_0.5_ derived from the experimental data is 1.5 mM. Comparing this data with those previously reported for SVCT2, profound differences are evidenced. Indeed the Km of the mitochondrial SVCT2 for ascorbate depends on cell types (U937, Raw 264.7) and medium composition, ranging from 8.4 and 27 μM (Fiorani et al., 2015), thus being some orders of magnitude different from the K_0.5_ of the herein described transporter. However, in other reports, the Km measured in intact mitochondria from HEK-293, using transport buffer designed to re-create the ionic conditions of the intracellular milieu, such as lower sodium concentrations, was 0.6 mM and a cooperative interaction of two ascorbate molecules with SVCT2 was described (Muñoz-Montesino et al., 2014). It has been proposed that SVCT2 may be responsible of ascorbate transport under altered metabolic conditions (Muñoz-Montesino et al., 2014) and justified the inconsistency between the low Km of mitochondrial SVCT2 and the high substrate concentration by the existence of mechanisms that regulate the transporter activity (Fiorani et al., 2015). The transport protein here described is sodium independent, it has a different molecular mass, pH dependence, and Km for ascorbate thus suggesting that it is diverse from SVCT2. The Mitochondrial Ascorbic acid Transporter-MAT is sodium independent, and with a Km of the same magnitude of intracellular vitamin C range concentration (Li et al., 2001), can indeed be involved in its translocation across the inner mitochondrial membrane under physiological conditions. Due to methodological reasons, related to its very low activity, MAT potato transporter has not been kinetically characterized in the present work. Indeed, since we have ascertained that it shares all the other biochemical tested features with the rat protein, we can argue that it might perform the same metabolic role. Also in plants, cytosolic ascorbic acid concentration largely varies among different tissues and metabolic state of the cells, and it is reported higher than mitochondrial, in the range of 20 mM out vs. 10 mM in (Szarka et al., 2013). Moreover mitochondrial ascorbate transport has been analyzed in other works, but the molecular determinants responsible of ascorbic acid transport across the plant inner mitochondrial membrane are until now elusive (Mandl et al., 2009; Szarka et al., 2013). In this work it has been for the first time demonstrated, both in mammal and plant mitochondrial membrane, the presence of Mitochondrial Ascorbate Transporter-MAT, which is different from SVCT2 and GLUT1. Transporters of the SLC25 familyalso known as the mitochondrial carrier family (MCF), are widespread in eukaryotes and characterized by a striking variety of transported solutes that widely vary in structure and size. Until now, 24 mitochondrial carrier subfamilies have been characterized based on substrate specificity and kinetic properties and at least 20 of them are “orphan” members of the SLC25 family, i.e., not yet identified (Palmieri 2013). Taking into accounts some features such as the molecular weight we can suppose that MAT is codified by one of this orphan genes. MAT, can be considered a new member joining the already recognized mitochondrial ascorbate transporters (Bánhegyi et al., 2014) showing different biochemical characteristics. In conclusion, the data reported in this work are the first example of reconstitution in proteoliposomes of MAT. In addition, the functional reconstitution of the ascorbate transporter described in this paper adds another piece to the puzzle that is the vitamin C transport across the inner mitochondrial membrane and should be a useful basis for further characterization of this protein at a molecular level.

## Author contributions

VS contributed conception and design of the study. NG contributed in revising the manuscript and interpretation of data for the work. SDL contributed in the isolation and purification of potato mitochondria and in revising the manuscript. LC performed SDS page experiments, contributed in revising the manuscript. EC performed the experiments and contributed in the isolation and purification of rat liver mitochondria. AT contributed conception and design of the study, wrote the manuscript and supervised the experiments.

### Conflict of interest statement

The authors declare that the research was conducted in the absence of any commercial or financial relationships that could be construed as a potential conflict of interest.

## Abbreviations

GLUT: glucose transporter
SVCT: sodium-dependent vitamin C transporter
DHA: dehydroascorbate
DTE: dithioerythritol
HTP: hydroxyapatite
SDS: sodium dodecylsulphate
p-OHMB: p-hydroxymercuribenzoate
NEM: N-ethylmaleimide
DIDS: 4,4′-diisothiocyano-2,2′-stilbene disulfonic acid
PLP: pyridoxal phosphate.
